# Renal tubular epithelium-targeted peroxisome proliferator-activated receptor-γ maintains the epithelial phenotype and antagonizes renal fibrogenesis

**DOI:** 10.18632/oncotarget.11811

**Published:** 2016-09-01

**Authors:** Min Zhao, Ying Chen, Guixia Ding, Ying Xu, Mi Bai, Yue Zhang, Zhanjun Jia, Songming Huang, Aihua Zhang

**Affiliations:** ^1^ Department of Nephrology, Nanjing Children's Hospital, State Key Laboratory of Reproductive Medicine, Nanjing Medical University, Nanjing, China; ^2^ Jiangsu Key Laboratory of Pediatrics, Nanjing Medical University, Nanjing, China

**Keywords:** PPAR-γ, TGF-β1, renal epithelial cells, epithelial cell phenotype, fibrosis, Pathology Section

## Abstract

Accumulating evidence suggests that loss of the renal tubular epithelial phenotype plays an important role in the pathogenesis of renal tubulointerstitial fibrosis. Systemic activation of peroxisome proliferator-activated receptor γ (PPAR-γ) has been shown to be protective against renal fibrosis, although the mechanisms are poorly understood. The present study aimed to define the role of renal tubular epithelium-targeted PPAR-γ in protection of the epithelial phenotype and the antagonism of renal fibrosis and to define the underlying mechanisms. In response to TGF-β1 challenge, PPAR-γ expression and activity in the renal proximal tubule epithelial cells (RPTECs) were significantly reduced, and the reduction was accompanied by decreased E-cadherin and elevated α-SMA, indicating a loss of the epithelial phenotype. Oxidative stress induced by TGF-β1 was shown to be attributed to the alteration of the epithelial phenotype and PPAR-γ inhibition. Activation of PPAR-γ by its agonists of rosiglitazone and 15d-PGJ2 or genetic overexpression of PPAR-γ prevented the loss of the epithelial phenotype induced by TGF-β1 in line with the inhibition of oxidative stress. To explore the role of PPAR-γ in renal tubular epithelial in antagonizing fibrogenesis, PPAR-γ was specifically deleted from RPTECs in mice. Following unilateral ureteral obstruction, the fibrosis was markedly deteriorated in mice with PPAR-γ invalidation in RPTECs. Treatment with rosiglitazone attenuated tubulointerstitial fibrosis and epithelial phenotype transition in WT but not proximal tubule PPAR-γ KO mice. Taken together, these findings identified an important role of renal tubular epithelium-targeted PPAR-γ in maintaining the normal epithelial phenotype and opposing fibrogenesis, possibly via antagonizing oxidative stress.

## INTRODUCTION

Renal fibrosis is an inevitable outcome of all types of progressive chronic kidney disease. During the development and progression of renal fibrosis, alteration of the epithelial phenotype of renal tubular cells could impair the epithelial integrity and cellular function, which might subsequently trigger the fibrotic response in the kidney [1, 2]. The phenotypic conversion of epithelial cells involves the loss of epithelial proteins, such as E-cadherin, zonula occludens-1 (ZO-1) and cytokeratin, and the acquisition of new mesenchymal markers including vimentin, α-smooth muscle actin (α-SMA), fibroblast-specific protein-1 (FSP1), interstitial matrix components type I collagen, and fibronectin [3]. The loss of cell adhesion is accompanied by cytoskeletal remodeling and morphological changes, resulting in tubular basement membrane disruption. Consequently, these cells possess the ability to migrate from the tubule basement membrane into the interstitium [4]. Moreover, emerging evidence suggested that pathological insult-activated renal tubular cells might stimulate the proliferation of fibroblast cells located in the tubulointerstitial region *via* the transduction of fibrogenic signals [5].

PPAR-γ is a ligand-activated transcription factor and belongs to the nuclear hormone receptor superfamily. PPAR-γ is highly expressed in adipose tissue and plays a crucial role in adipocyte differentiation [6]. Moreover, PPAR-γ regulates a diverse spectrum of physiological and pathological processes including insulin sensitivity, lipid metabolism, inflammation, immune regulation, and extracellular matrix balance [7, 8]. In the kidney, PPAR-γ is expressed in all types of glomerular, tubular, and tubulointerstitial cells and is of importance in the pathogenesis of many types of kidney diseases [8, 9]. In the past decade, accumulating evidence from systemic activation of PPAR-γ showed an antifibrotic effect in CKD models [10-12]. However, the cell-specific role of PPAR-γ in modulating renal fibrosis remains uncertain.

In the present study, employing proximal tubule-specific PPAR-γ knockout (KO) mice and the PPAR-γ agonists rosiglitazone (RGZ) and 15-deoxyprostaglandin J2 (15d-PGJ2), we studied the role of renal tubular epithelium-targeted PPAR-γ in regulating the fibrotic response *in vitro* and *in vivo* and the potential mechanisms.

## RESULTS

### TGF-β1 suppressed PPAR-γ expression and activity in HK-2 cells

**Figure 1:**
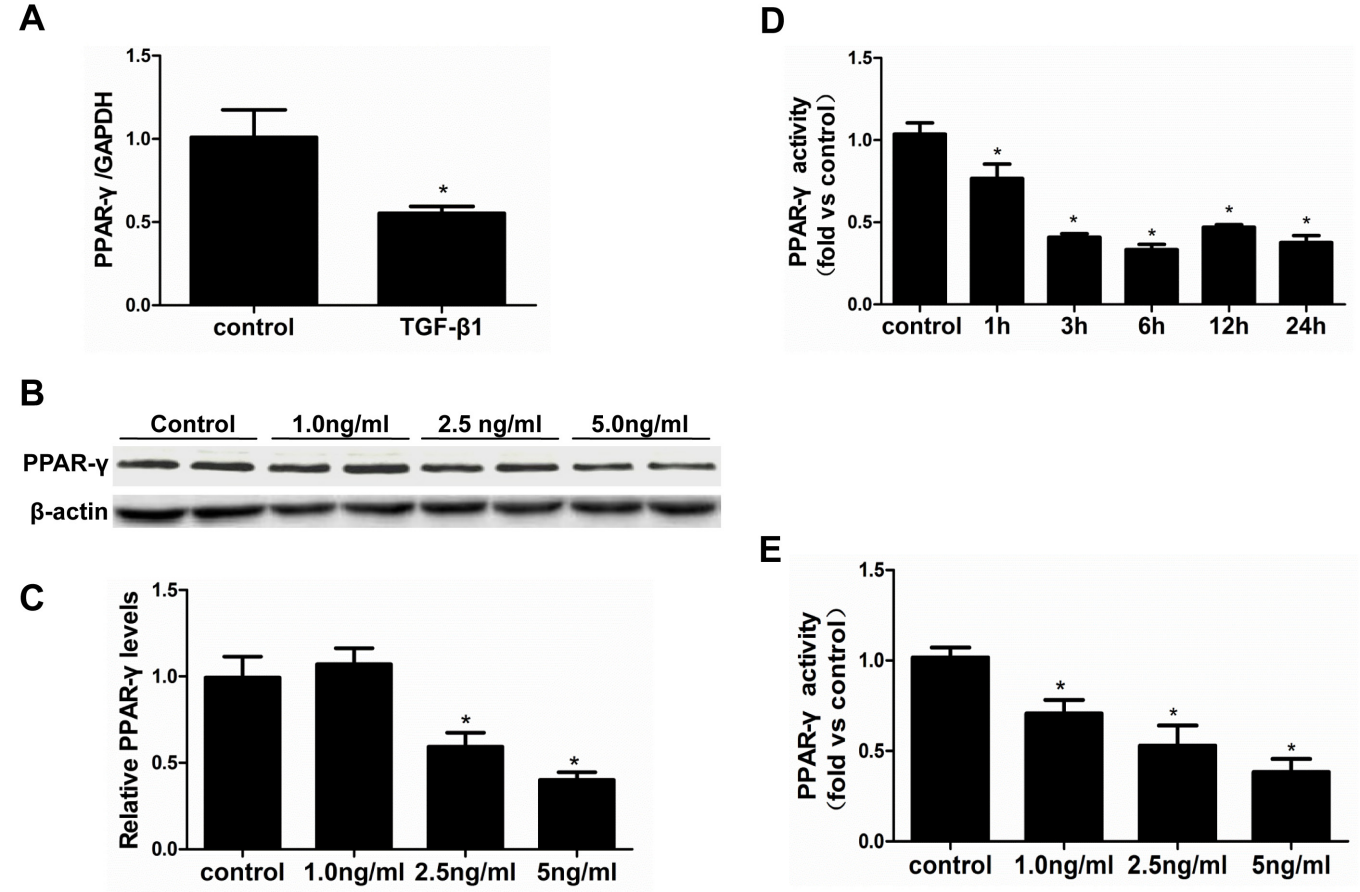
TGF-β1 suppressed PPAR-γ expression and activity in HK-2 cells The cells were grown on 6-well plates until 80% confluence and then treated with vehicle or TGF-β1 for 24h. **A.** qRT-PCR analysis of PPAR-γ after treated with TGF-β1 (5ng/ml). **B.** Representative immunoblots. **C.** Densitometric analysis of PPAR-γ protein expression. **D.** & **E.** PPAR-γ transcription factor assay kit was used to detect PPAR-γ activity at different time points **D.** and different doses **E.**. Values are means ± SE, *n* = 6 in each group, * *P* < 0.05 *vs*. control.

HK-2 cells were used in all vitro experiment. First, we evaluated the influence of TGF-β1 on PPAR-γ expression and activity. After treatment with TGF-β1 (5 ng/ml) for 24 h, PPAR-γ mRNA expression was significantly decreased as determined by qRT-PCR (Figure 1A). Similarly, PPAR-γ protein expression was reduced in a dose-dependent manner (1.0, 2.5, 5.0 ng/ml) (Figure 1B & 1C). TGF-β1 strikingly inhibited PPAR-γ activity in time- and dose-dependent manners as detected by a PPAR-γ transcription factor assay kit (Figure 1D & 1E). These data indicate a potent role of TGF-β1 in suppressing PPAR-γ expression and activity.

### Oxidative stress mediated the TGF-β1 effect on PPAR-γ reduction and cellular phenotype alteration

**Figure 2:**
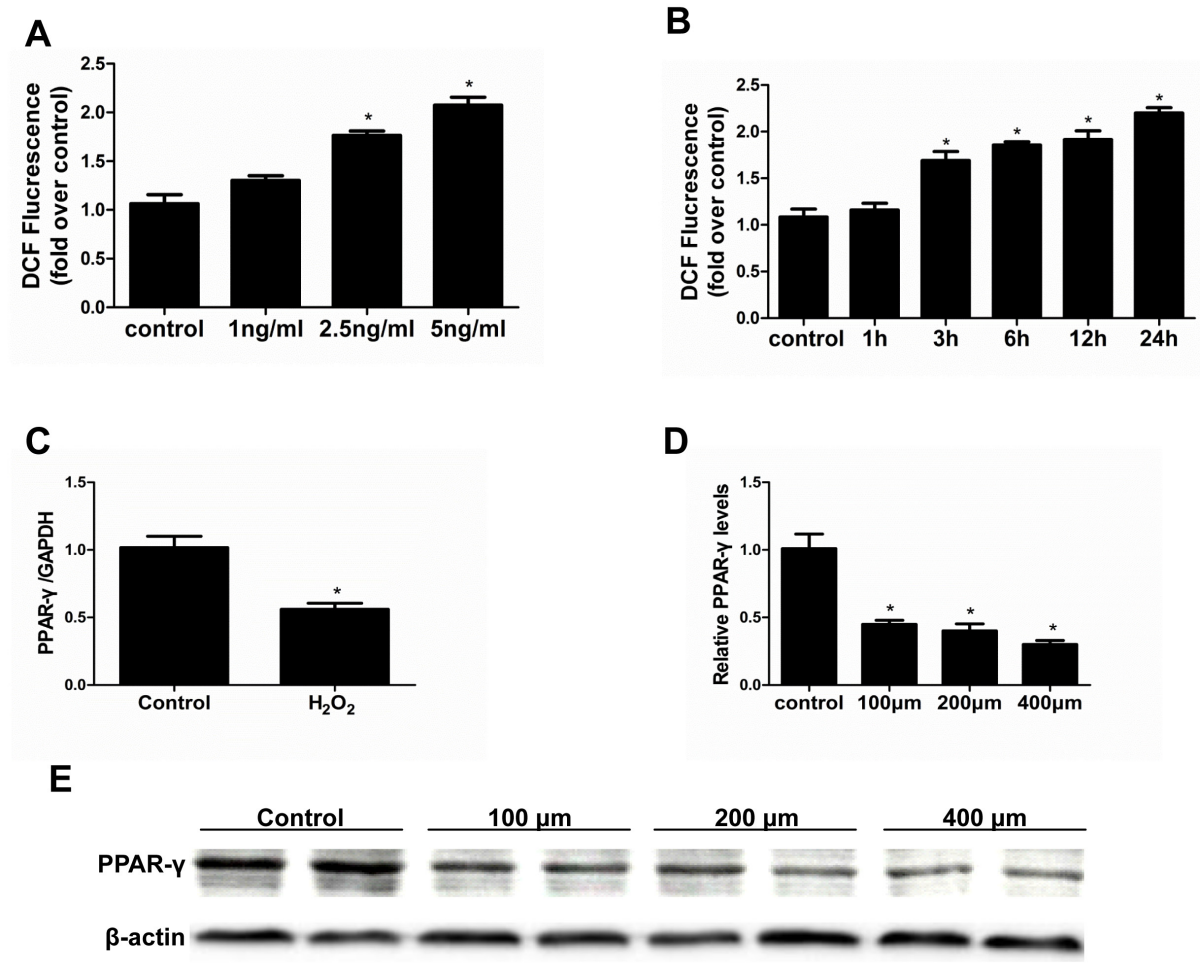
Effect of H_2_O_2_ administration on PPAR-γ expression Cells were grown on 6-well plates until 80% confluence and then treated with TGF-β1 (0, 1, 2.5, 5 ng/ml) for 2 hours or treated with vehicle or H_2_O_2_ (200 μm) **A.** or cells were treated with TGF-β1 (5ng/ml) at different time points (0, 1, 3, 6, 12, 24h) **B.** then ROS levels were analyzed by flow cytometry. **C.** mRNA expression of PPAR-γ was detected by qRT-PCR. **D.** Densitometric analysis of PPAR-γ expression. **E.** Western-blot for PPAR-γ expression Values are means ± SE, *n* = 6 in each group, * *P* < 0.05 *vs*. control.

Oxidative stress has extensive actions in modulating physiological and pathological responses. We tested the potential of oxidative stress in mediating the TGF-β1 effect on PPAR-γ reduction and cellular phenotype alteration. FH_2_O_2_ similarly mimicked Figure 2C-2E).

**Figure 3:**
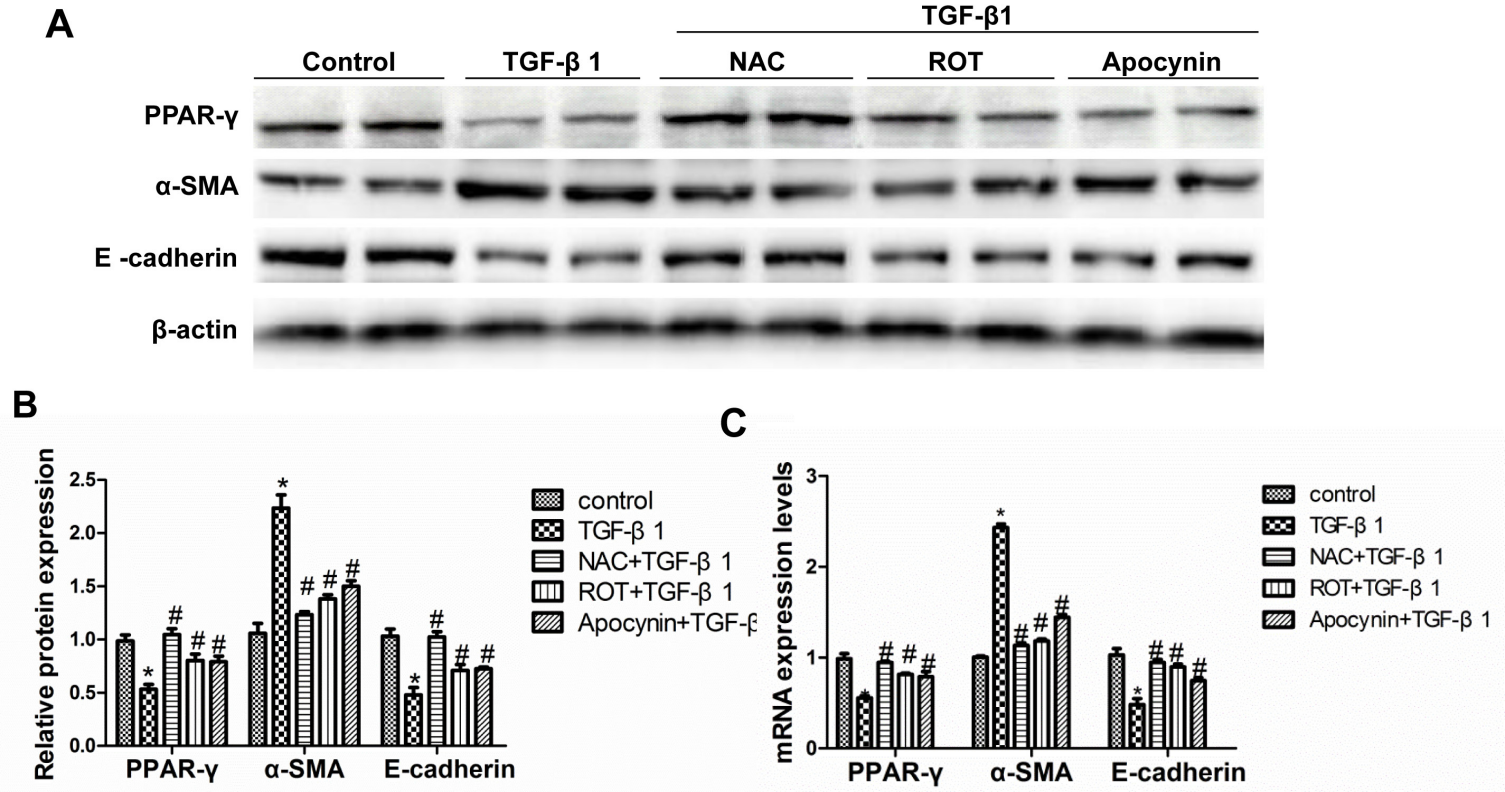
Inhibition of TGF-β1-induced oxidative stress restored PPAR-γ downregulation and the alteration of cellular phenotype The cells were grown on 6-well plates until 80% confluence and then pretreated with NAC (5 mM), ROT (10 μmol/L), and Apocynin (500 μmol/L) for 30 min followed by TGF-β1 treatment for another 24h. **A.** Western-blot for PPAR-γ, α-SMA and E-cadherin expression. **B.** Densitometric analysis of the immunoblots for α-SMA and E-cadherin. **C.** qRT-PCR analysis of PPAR-γ, α-SMA and E-cadherin mRNA expression. Values are means ± SE, *n* = 6 in each group, * *P* < 0.05 *vs*. Control, # *P* < 0.05 *vs*. TGF-β1-treated group.

To define the contribution of different sources of ROS on the suppression of PPAR-γ, the mitochondrial inhibitor rotenone, NADPH oxidase inhibitor apocynin, and a nonspecific antioxidant N-acetyl-L-cysteine (NAC) were applied to treat the cells. As shown in Figure 3A-3C, NAC significantly blocked TGF-β1-induced PPAR-γ reduction, whereas the mitochondrial respiratory chain complex I inhibitor rotenone and the NADPH oxidase inhibitor apocynin partially prevented TGF-β1-induced PPAR-γ downregulation. The increase of α-SMA and decrease of E-cadherin were abolished by NAC, contrasting to a partial effect of rotenone or apocynin. indicating that mitochondria- and NADPH oxidase-derived ROS contributed to this pathological process.

### PPAR-γ activation prevented the cellular phenotype alteration induced by TGF-β1

**Figure 4:**
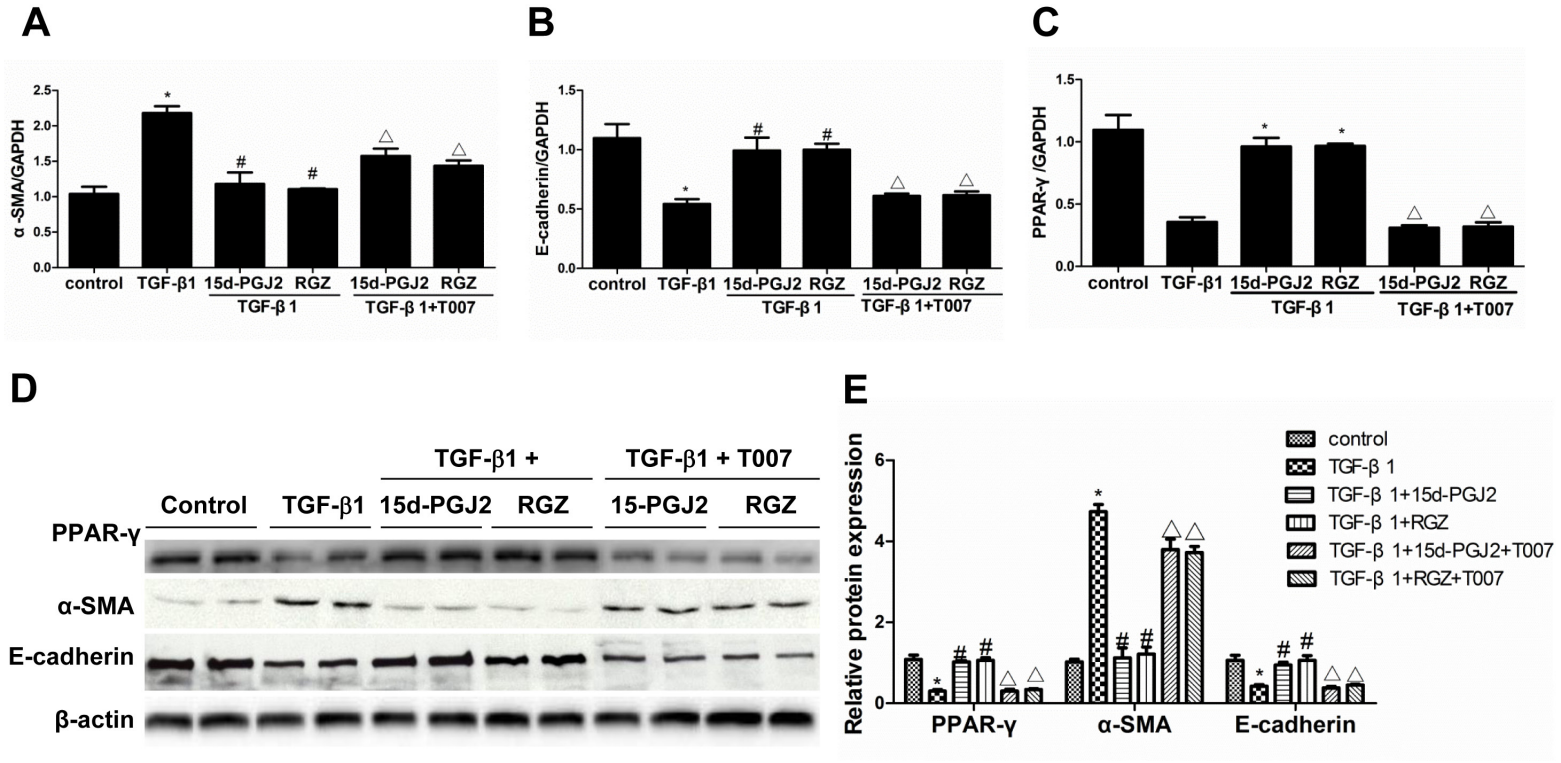
PPAR-γ activation inhibited TGF-β1-induced alteration of cellular phenotype The cells were grown in 6-well plates until 80% confluence and then pretreated with RGZ or 15d-PGJ2 for 30 min followed by TGF-β1(10 ng/ml) treatment for 72h in the presence or absence of PPAR-γ inhibitor T0070907. **A.**-**C.** qRT-PCT analysis of α-SMA, E-cadherin, and PPAR-γ mRNA expression. **D.** Western blots of PPAR-γ, α-SMA, and E-cadherin. **E.** Densitometric analysis of PPAR-γ, α-SMA, and E-cadherin. Values are means ± SE. Values are means ± SE, *n* = 6 in each group, * *P* < 0.01 *vs*. control group, # *P* < 0.01 *vs*. TGF-β1-treated group, *P < 0.01 *vs*. PPAR-γ agonists (RGZ or 15d-PGJ2)-treated group.

**Figure 5:**
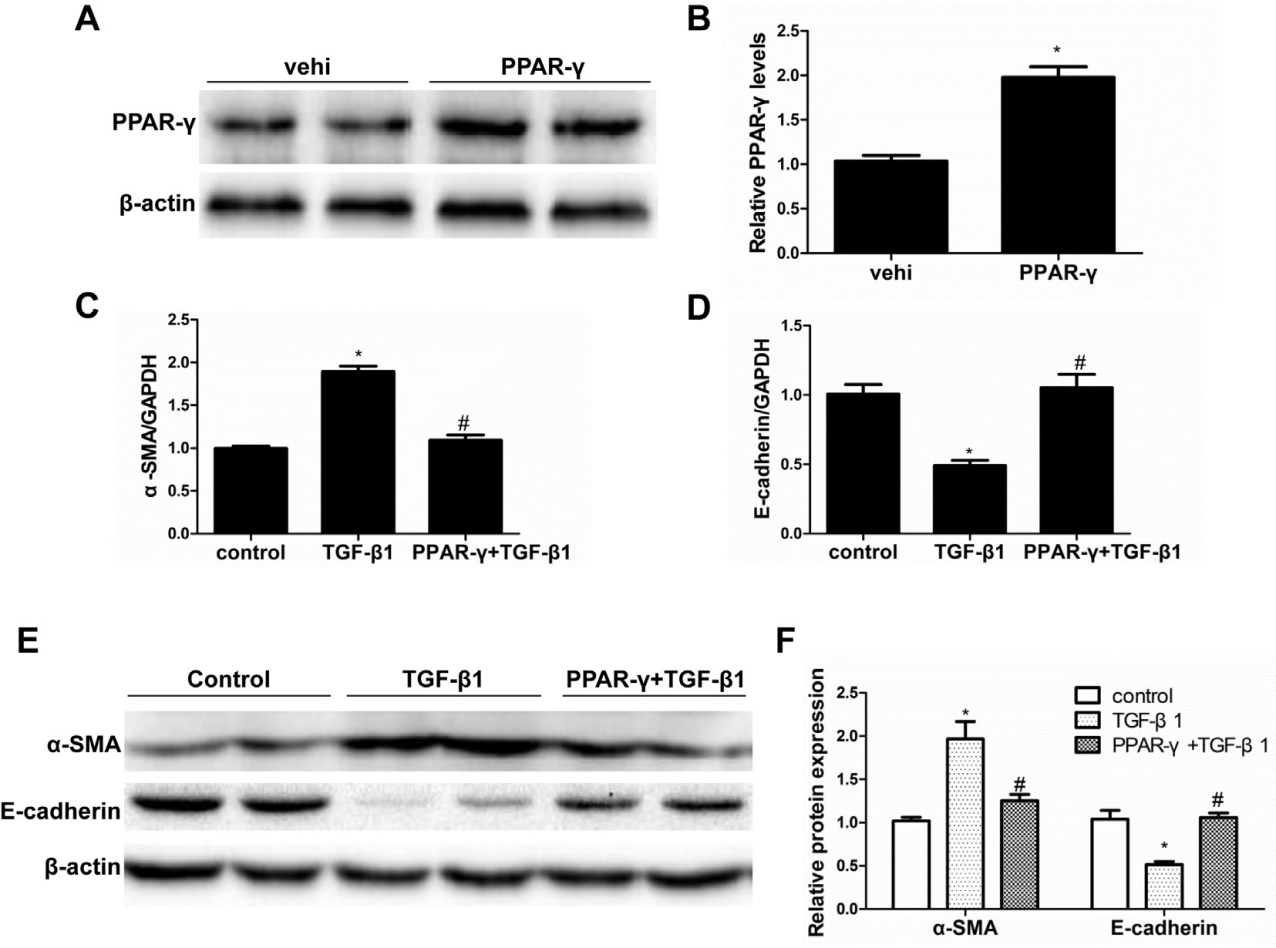
PPAR-γ overexpression blocked TGF-β1-induced phenotypic alteration **A.** Western blotting analysis of PPAR-γ in HK-2 cells transfected with PPAR-γ or empty vector (vehicle). **B.** Densitometric analysis of PPAR-γ. **C.** qRT-PCR analysis of α-SMA. **D.** qRT-PCR analysis of E-cadherin. **E.** Western blots of E-cadherin and α-SMA. **F.** Densitometric analysis of E-cadherin and α-SMA. Values are means ± SE, *n*= 6 in each group, * *P* < 0.05 *vs*. Control, # *P* < 0.05 *vs*. TGF-β1-treated group.

RGZ and 15d-PGJ2 were administered to the cells with or without the PPAR-γ inhibitor T0070907. As shown in Figure 4A-4E, RGZ and 15d-PGJ2 restored the induction of α-SMA and the reduction of E-cadherin, which was abolished by a specific PPAR-γ inhibitor T0070907. To rule out a PPAR-γ-independent effect, we overexpressed PPAR-γ in HK-2 cells (Figure 5A-5B). As expected, PPAR-γ overexpression showed a similar effect as PPAR-γ agonists in protecting the epithelial phenotype in HK-2 cells (Figure 5C-5F).

### Activation of PPAR-γ inhibited TGF-β1-induced ROS generation

**Figure 6:**
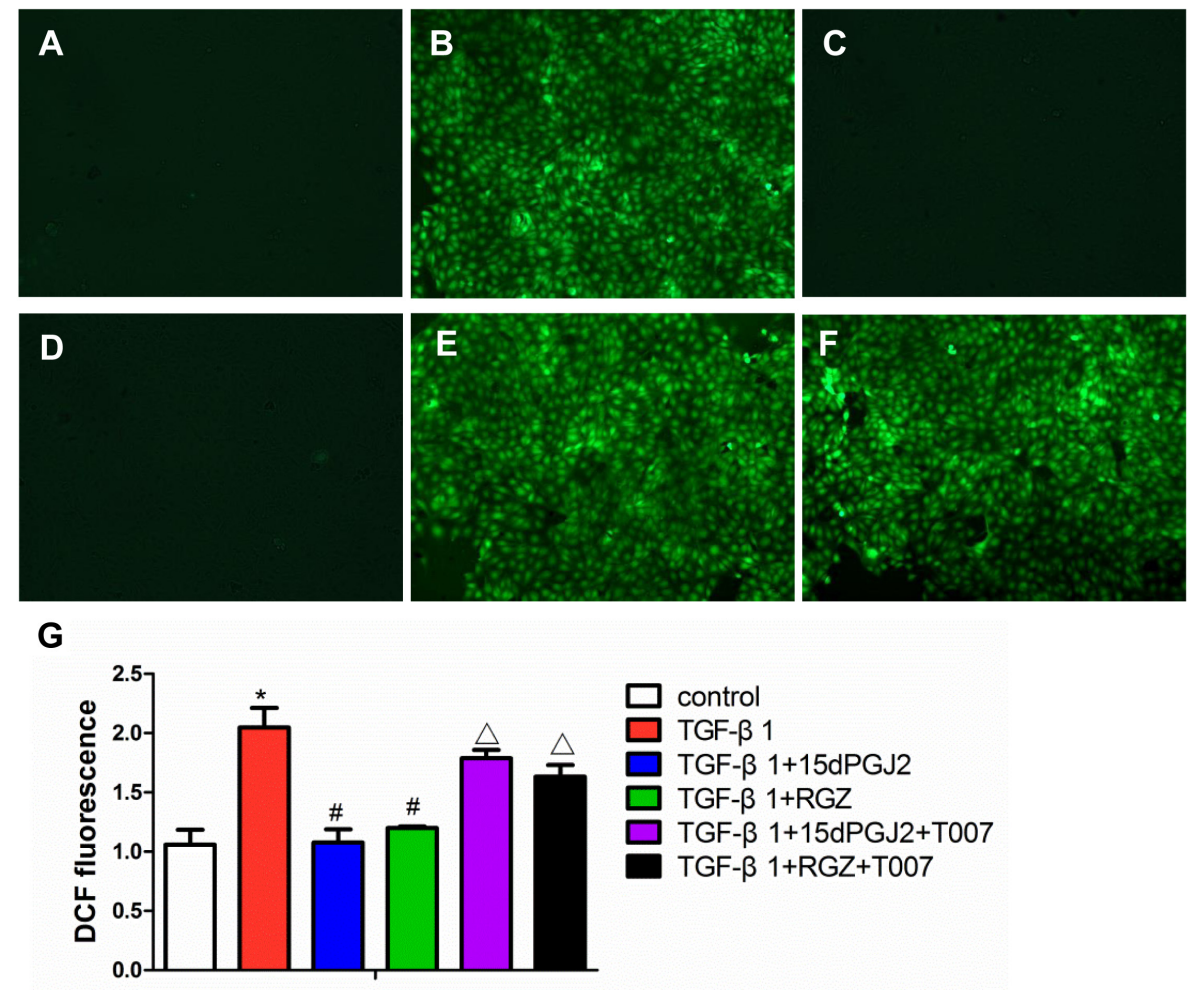
Activation of PPAR-γ inhibited TGF-β1-induced ROS production Confluent HK-2 cells in chamber slides were pretreated with PPAR-γ agonists and inhibitors for 30 min and then exposed to TGF-β1 for another 2h in the presence of DCFDA. **A.** control. **B.** 10 ng/ml TGF-β1. **C.** 10 ng/ml TGF-β1 plus 15d-PGJ2. **D.** 10 ng/ml TGF-β1 plus RGZ. **E.** 10 ng/ml TGF-β1 plus 15d-PGJ2 and T0070907. **F.** 10 ng/ml TGF-β1 plus RGZ and T0070907. **G.** Quantitation of DCF fluorescence. Fluorescence was quantified using FLUOstar OPTIMA. Values are means ± SE, *n* = 6 in each group, * *P* < 0.05 *vs*. Control, # *P* < 0.05 *vs*. TGF-β1-treated group. ## *P* < 0.05 *vs*. TGF-β1plus RGZ or 15d-PGJ2 groups.

In this experiment, we examined PPAR-γ action in modulating ROS production. induced ROS production was entirely abolished by treatment with RGZ or 15d-PGJ2. However, such an effect was remarkably blunted by a PPAR-γ antagonist (Figure 6A-6G).

### Proximal tubule epithelium-specific ablation of PPAR-γ aggravated renal fibrosis

**Figure 7:**
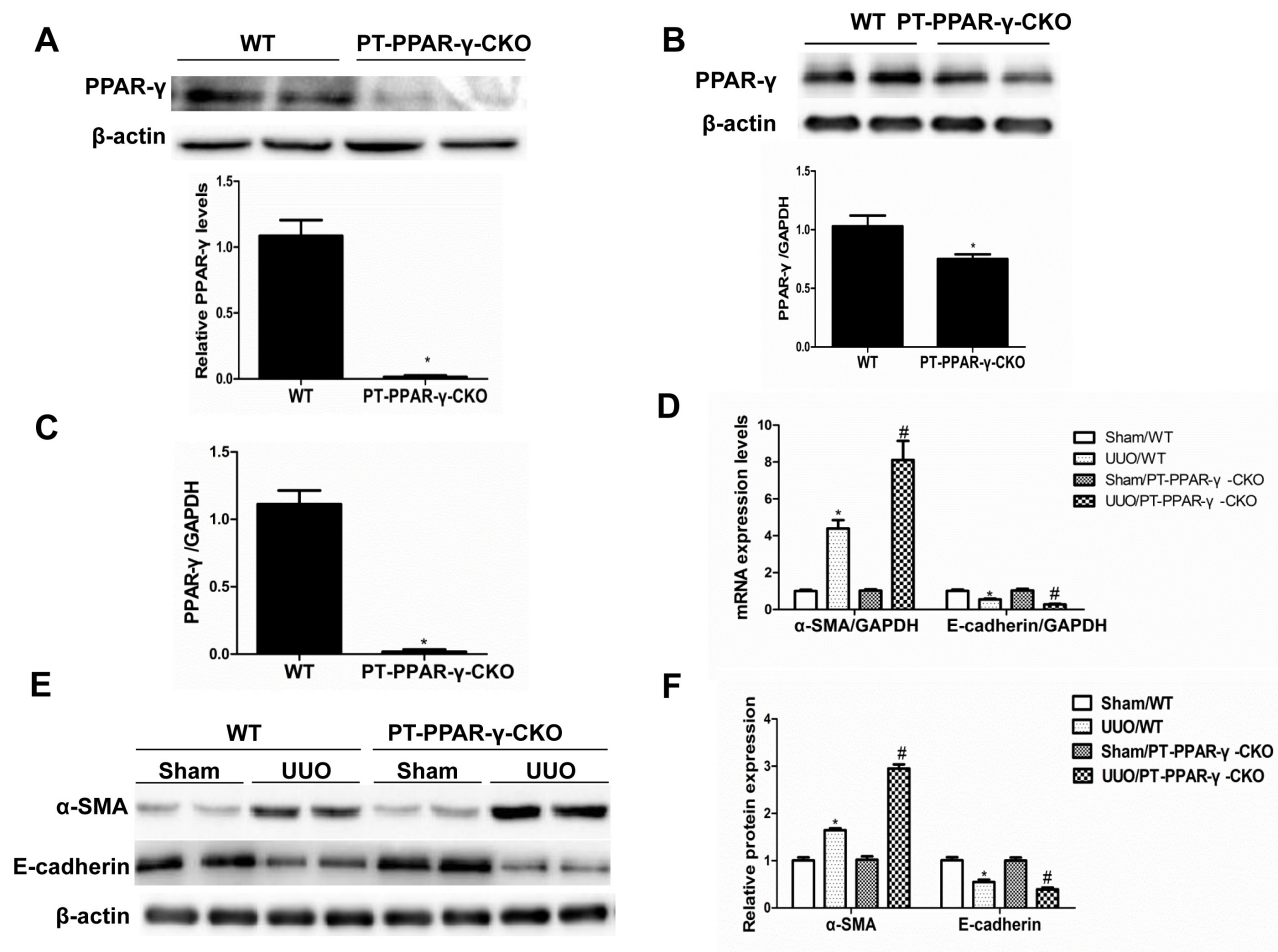
Proximal tubule epithelium-specific ablation of PPAR-γ aggravated the alteration of epithelial phenotype following UUO **A.** Western blotting analysis of PPAR-γ protein expression in primary proximal tubule cells from WT and PT-PPAR-γ-CKO mice. **B.** Whole kidney PPAR-γ expression from WT and PT-PPAR-γ-CKO mice. **C.** PPAR-γ mRNA expression of primary proximal tubule cells from WT or PT-PPAR-γ-CKO mice. **D.** qRT-PCR analysis of E-cadherin and α-SMA. **E.** Western blotting analysis of E-cadherin and α-SMA. **F.** Densitometric analysis of E-cadherin and α-SMA. Values are means ± SE, *n* = 6 in each group, * *P* < 0.01 *vs*. Sham/WT, # *P*< 0.01 *vs*. UUO/WT.

**Figure 8:**
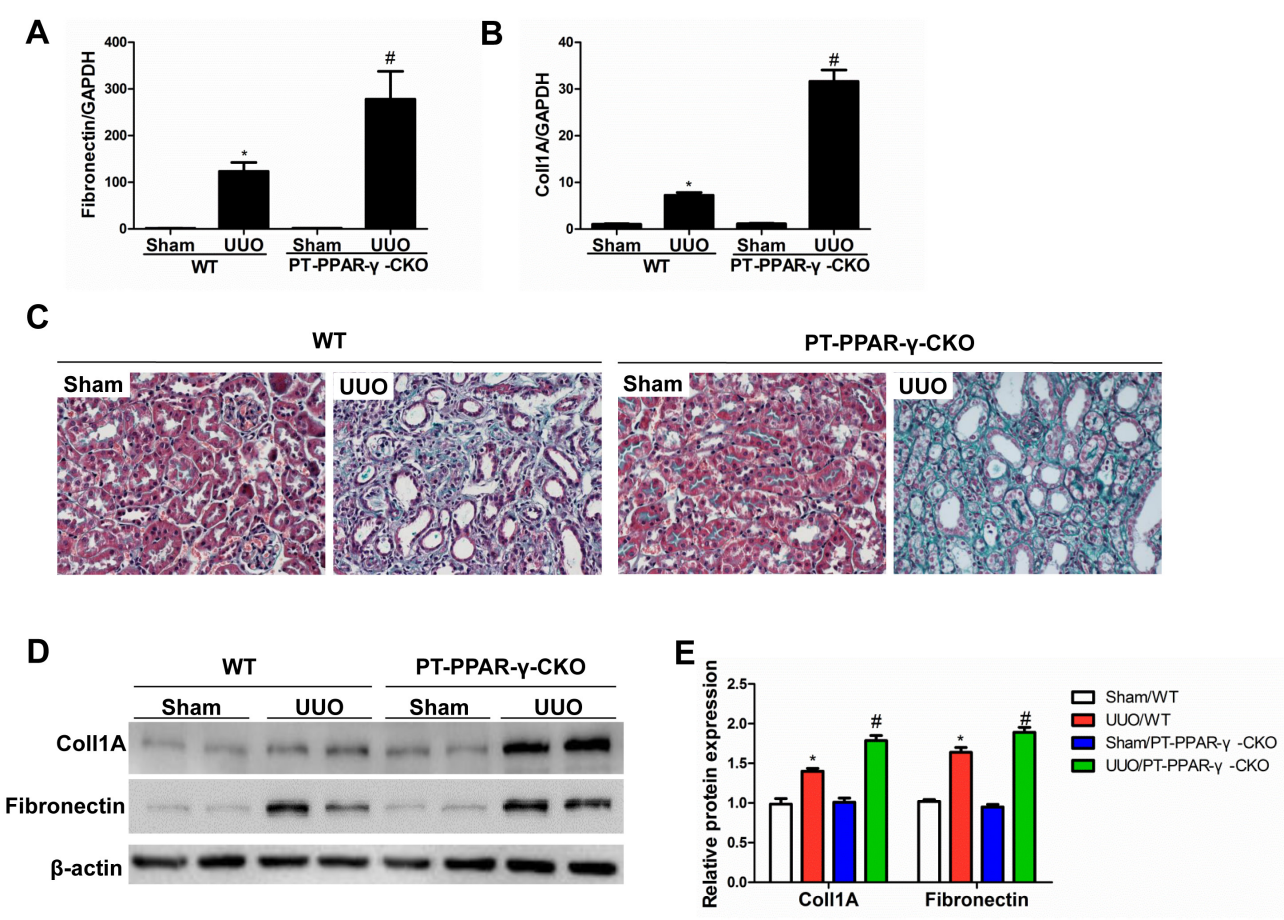
Proximal tubule epithelium-specific ablation of PPAR-γ aggravated renal fibrosis **A.** qRT-PCR analysis of fibronectin. **B.** qRT-PCR analysis of collagen-1. **C.** Representative images of Masson's trichrome staining. **D.** Western blotting analysis of TGF-β1, collagen-1 and fibronectin. **E.** Densitometric analysis of collagen-1 and fibronectin. Values are means ± SE, *n* = 6 in each group, * *P* < 0.01 *vs*. Sham/WT, # *P* < 0.01 *vs*. UUO/WT.

To investigate the *in vivo* role of renal epithelial PPAR-γ in opposing fibrogenesis, we generated mice with specific PPAR-γ deletions in the proximal tubular epithelial cells (Figure 7A-7C). Following a 14-day UUO, the PT-PPAR-γ-CKO mice lost more of the epithelial phenotype, shown by a greater elevation of The PT-PPAR-γ-CKO mice developed more severe tubulointerstitial fibrosis than the WT controls, as evidenced by the greater deposition of extracellular matrix proteins (Figure 8A-8E). *in vivo*

### Rosiglitazone attenuated renal fibrosis in WT mice but not in PT-PPAR-γ-CKO mice

**Figure 9:**
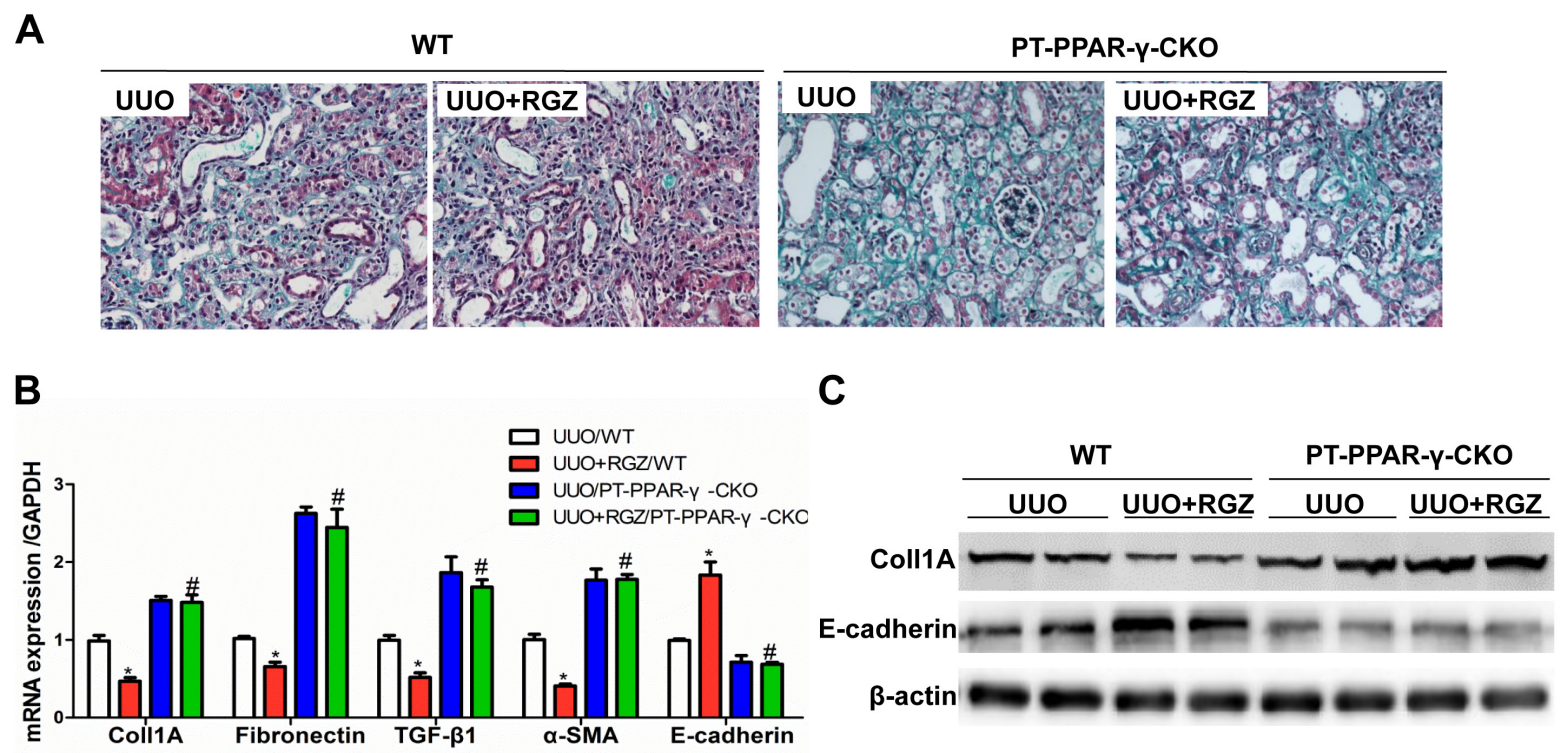
RGZ treatment attenuated renal fibrosis in WT mice but not in PT-PPAR-γ-CKO mice **A.** Representative images of Masson's trichrome staining. **B.** qRT-PCR analysis of fibronectin, collagen-1, TGF-β1, E-cadherin, and α-SMA. **C.**. Western blotting analysis of collagen-1 and E-cadherin. Values are means ± SE, *n* = 6 in each group, * *P* < 0.01 *vs*. Sham/WT, # *P* < 0.01 vs. UUO/WT.

Along the whole nephron, PPAR-γ is expressed in glomeruli, proximal tubules, and distal nephron. To further figure out the contribution of proximal tubular PPAR-γ in antagonizing fibrotic response in UUO model, we treated UUO WT and UUO PT-PPAR-γ-CKO mice with a specific PPAR-γ agonist RGZ. As shown in Figure 9, RGZ could ameliorate fibrosis and epithelial phenotype transition in WT mice but not in KO mice, suggesting that PPAR-γ in proximal tubules but not in other nephron segments or cell types played a key role in opposing renal fibrotic response in this experimental setting.

## DISCUSSION

Renal fibrosis is a common outcome of all types of CKDs. Although efforts have been made in the field, the pathogenic mechanisms remain uncertain. There is still no effective therapy against renal fibrosis. In recent years, PPAR-γ has drawn substantial attention for the treatment of kidney diseases including CKD-related renal fibrosis [7, 13]. However, the detailed molecular mechanisms and the role of cell-specific PPAR-γ in antagonizing fibrosis are unknown. In this study, we demonstrated that TGF-β1 suppressed PPAR-γ expression and activity *via* enhanced oxidative stress, and activated the PPAR-γ protected renal tubular cells *via* suppressing oxidative response. In agreement with the *in vitro* results, the conditional deletion of PPAR-γ in the tubular epithelium aggravated renal fibrosis and the tubular epithelial phenotype transition.

Accumulating evidence demonstrated that TGF-β1 serves as a principal mediator of fibrogenesis in the majority of organs [14, 15]. TGF-β1 alters the cellular phenotype and promotes renal fibrosis *via* its downstream components of Smads [2, 16]. TGF-β1 attenuates myocardial PPAR-γ expression in pressure overload-induced cardiac fibrosis and remodeling in mice [17]. Consistent with the previous findings, TGF-β1 treatment in renal epithelial cells caused typical changes including loss of E-cadherin, and de novo expression of α-SMA, indicating a fibrotic phenotype. In agreement with the alteration of the cellular phenotype, PPAR-γ was down-regulated by TGF-β1, suggesting a potential role of PPAR-γ in opposing the TGF-β1 effect.

PPAR-γ is a ligand-activated transcription factor and belongs to the nuclear hormone receptor superfamily. Ligands for PPAR-γ include a variety of natural and synthetic compounds. We demonstrated that TGF-β1 dose-dependently decreased PPAR-γ expression and increased oxidative stress. The formation of ROS is a natural consequence of aerobic metabolism and is integral for maintaining tissue oxygen homeostasis [18]. ROS are also important in the coordination and activation of numerous signal transduction pathways [19]. However, excessive ROS could cause cell damage and organ injury. In cultured HK-2 cells, TGF-β1-induced ROS was blocked by RGZ (a synthetic PPAR-γ agonist) and 15d-PGJ2 (a natural PPAR-γ agonist), demonstrating an anti-oxidative role of PPAR-γ. We determined that H_2_O_2_,TGF-β1 effect in suppressing PPAR-γ and altering the epithelial phenotype. These data suggest a mutual antagonism between PPAR-γ and TGF-β1/ROS axis in renal epithelial cells. Moreover, using the mitochondrial inhibitor rotenone and the NADPH oxidase inhibitor apocynin, we showed that mitochondria- and NADPH oxidase-derived ROS participated in this pathological process. To rule out the nonspecific effect of PPAR-γ agonists, we performed another experiment by genetically overexpressing PPAR-γ in HK-2 cells. In this experiment, we observed a similar effect as RGZ and 15d-PGJ2 in the prevention of cellular phenotypic changes. PPAR-γ ligands including rosiglitazone, ciglitazone, and troglitazone and other members of TZD family, are popular used in gypoglycemic therapy for type 2 diabetes. PPAR-γ agonists could ameliorate renal fibrotic lesions in chronic kidney diseases including diabetic nephropathy. Studies in proximal epithelial cells, such as NRK52E and HK2 cell lines, shown PPAR-γ ligands play protective role on anti-fibrosis and TGF-β/Smads signaling is considered the most important pathway in EMT and fibrosis. In renal tubular epithelial cells, Li R et al shown curcumin blocked TGF-β1-induced EMT *via* ERK-dependent and then PPAR-γ-dependent pathway but not Smad2 or Smad3 [20]. Wei et al found CDDO, a synthetic PPAR-γ agonist, attenuates fibrogenesis by antagonistically targeting canonical TGF-β/Smad and Akt signaling in a PPAR-γ-independent manner [21]. In human lung fibroblasts, AA Kulkarni et al shown that PPAR-γ ligands play an anti-fibrotic role through inhibiting TGF-β1-induced Akt phosphorylation, the inhibition is independent of MAPK-p38 and PTEN but is dependent on TGF-β1-induced phosphorylation of FAK, a kinase that acts upstream of Akt [22].

PPAR-γ is expressed in multiple renal cell type including the cultured glomerular mesangial cells, podocytes, proximal epithelial cells [23, 24], and dominantly expressed in the collecting system of the mammalian urinary tract, including connective renal tubules and collecting ducts. The PPAR-γ ligand TZDs affect extracellular matrix and cytoskeleton remodeling and metabolic processes of the kidney collecting duct epithelia including carbohydrate, lipid metabolism [25, 26]. Studies shown activation of PPAR-γ in the distal collecting system causes severe water retention, a common side effect after thiazolidinedione treatment [27, 28]. Zhang H et al shown collecting duct-specific deletion of PPAR-γ blocks thiazolidinedione-induced fluid retention, rosiglitazone stimulated sodium transport in primary cultures of CD cells expressing PPAR-γ and not in cells lacking this receptor [29]. However, whether PPAR-γ in connective renal tubules and collecting ducts plays a similar role as proximal tubule in the pathogenesis of renal fibrosis have not be reported. In our study, rosiglitazone diminished renal fibrosis in WT mice, but not in mice with specific proximal epithelial cell PPAR-γ deletion.

Recent studies identified that PPAR-γ agonists could inhibit renal fibroblast activation and the fibrosis of tubule cells [12, 30, 31]. To further evaluate if epithelium-targeted PPAR-γ plays a role in maintaining the normal cellular phenotype and antagonizing fibrosis, we specifically deleted PPAR-γ in the proximal tubular cells of mice. Following 14 days of UUO, the KO mice exhibited a remarkable deterioration in the tubulointerstitial fibrosis as evidenced by the greater deposition of extracellular matrix. The loss of E-cadherin and the gain of α-SMA were greater in the KO mice, suggesting a more serious change in the epithelial phenotype. These important data offered the first evidence showing an *in vivo* role of renal tubular PPAR-γ in opposing renal fibrosis and maintaining normal cellular phenotypes possibly *via* antagonizing TGF-β signaling in both epithelium and fibroblasts.

Employing genetic and pharmacological strategies, we demonstrated an essential role of renal tubular PPAR-γ in protecting kidneys against fibrosis and cellular phenotype alteration possibly *via* antagonizing TGF-β1 and TGF-β1-induced oxidative stress. The findings enriched our understanding of the PPAR-γ effect on modulating kidney function and the pathogenesis of renal fibrosis and also provided novel insights into CKD therapies by targeting renal tubular molecules and signaling pathways.

## MATERIALS AND METHODS

### Reagents and antibodies

Recombinant human transforming growth factor-β1 (TGF-β1, Cat#: 100-21) was from PeproTech (Rocky Hill, NJ), N-acetyl-L-cysteine (Cat#: A7250), apocynin (Cat#: A108109), rotenone (Cat#: R8875), RGZ (Cat#: R2408), T0070907 (Cat#: T8703, inhibitor of PPAR-γ), 2′,7′-dichlorofluoresceindiacetate (DCFDA, Cat#: D6883), and 15-deoxyprostaglandin J2 (Cat#: D8440), were from sigma (St. Louis, MO). Rat monoclonal anti-E-cadherin (Cat#: ab76055) and anti-α-SMA (Cat#: ab5694) antibodies, rabbit polyclonal anti-PPAR-γ antibody (Cat#: ab19481) were from abcam (Cambridge, MA). PPAR-γ transcription factor assay kit (Cat#: KA1358) was from abnova (Walnut, CA). PPAR-γ plasmid (Plasmid #8895) was from addgene (Cambridge, MA). Proteinase K (Cat#: P2308) was from sigma (St. Louis, MO).

### Human kidney-2 (HK-2) cells

HK-2 cells, the immortalized human proximal tubular cell line, were grown in keratinocyte serum-free media (KSFM) supplemented with bovine pituitary extract and epidermal growth factor (Invitrogen). The cells were grown at 37°C in a humidified 5% CO_2_ incubator and subcultured at 60-80% confluence using 0.05% trypsin-0.02% EDTA (Invitrogen).

### Transient transfection of HK-2 cells with PPAR-γ plasmid

Cells grown to 40% confluence in six-well plates were transiently transfected with PPAR-γ plasmid or vector with lipofectamine 2000 (Invitrogen) according to the manufacturer's protocol. Cells were transfected with PPAR-γ plasmid for 24 h before treatment with TGF-β1.

### Animals

Mice with the floxed PPAR-γ gene and kap-cre mice were from Jackson Laboratory (Bar Harbor, ME) and had a genetic background of C57/B6J. In brief, 12-14-week-old male mice weighing 25-30 g were used in the experiment. All mice were maintained on a 12-h light-dark cycle in a temperature-controlled room and were fed standard rodent chow. The animal study protocols were reviewed and approved by the Institutional Animal Care and Use Committee at Nanjing Medical University, China (No. 20090053).

### Isolation of DNA from mouse tails

Cut off 0.2cm tail from 2- to 3-week old mice and put it into the tube with 300μl tail lysate including Proteinase K (10mg/ml). After incubation overnight at 55 degrees in water bath, 600μl 100% EtOH was added and vortexed vigorously. Then the sample was centrifuged by 12,000g for 5 min at room temperature. After removing the supernatant, the DNA pellet was rinsed with 70% EtOH followed by 12,000g centrifugation for 5 min at room temperature. Then the supernatant was removed and the DNA pellet was allowed to air-dry for 5-10 min.

### Proximal tubule-specific deletion of PPAR-γ in mice and genotyping

To generate the proximal tubule-specific deletion of PPAR-γ in mice, homozygous mice with PPAR-γ ^flox/flox^ were crossed with kap-cre mice to obtain PPAR-γ^flox/+^ kap-cre(+) mice. Then, the PPAR-γ^flox/+^ kap-cre(+) mice were crossed with PPAR-γ ^flox/flox^ mice to obtain PPAR-γ^flox/flox^ kap-cre(+) male mice (PT-PPAR-γ-CKO). All male Cre transgene negative littermates served as controls (wild type, WT). A routine PCR protocol was used for genotyping tail DNA with the following primer pairs: PPAR-γ genotyping, forward: 5′-TGTAATGGAAGGGCAAAAGG-3′ and reverse: 5′-TGGCTTCCAGTG CATAAGTT-3′, which yielded 250- and 200-bp bands, respectively, for the floxed and wild-type alleles; Cre transgene genotyping, forward: 5′-AGATGCCAGGACATCAG GAACCTG-3′ and reverse: 5′-ATCAGCCACACCAGACACAGAGATC-3′, which generated a 200-bp fragment.

### Primary culture of proximal tubule cells

Primary proximal tubular epithelial cells were isolated and cultured as previous described [13]. WT and PT-PPAR-γ-CKO mice (20-30 days) were sacrificed, and the renal cortices were sliced into pieces ∼1 mm wide in ice-cold dissection solution (DS) (HBSS with in mmol/l: 10 glucose, 5 glycine, 1 alanine, 15 HEPES, pH 7.4 and osmolality 325 mosmol/kgH_2_O), followed by collagenase solution [DS with 0.1% (wt/vol) type-2 collagenase and 96 μg/ml soybean trypsin inhibitor] digestion for 30 min at 37°C. The supernatant was then sieved through two nylon sieves (pore size 250-μm and 80-μm). The longer PT fragments remained in the 80-μm sieve and were resuspended by flushing the sieve in the reverse direction with warm DS (37°C) containing BSA 1% (wt/vol). The PTs in the BSA solution were centrifuged for 5 min at 170 g, washed, and then resuspended in the appropriate amount of culture medium: 1:1 DMEM/F12 without phenol red and supplemented with heat-inactivated FCS 1%, HEPES 15 mmol/l, L-glutamine 2 mmol/l, hydrocortisone 50 nmol/l, insulin 5 g/ml, transferrin 5 μg/ml, selenium 50 nmol/l, sodium pyruvate 0.55 mmol/l, 100× Nonessential amino acids 10 ml/l, penicillin 100 IU/ml and streptomycin 100 μg/ml buffered to pH 7.4 and osmolality of 325 mOsmol/kgH_2_O. The PT fragments were seeded onto collagen-coated permeable PTFE-filter supports and left unstirred for 48 h at 37°C and 95% air/5% CO_2_ in a standard humidified incubator (Jouan, Winchester, VA). The culture medium was then changed for the first time. The medium was replaced every 2 days. After 7 days, the cell cultures were organized as a confluent monolayer.

### Animal model of unilateral ureteral obstruction (UUO)

Male PT-PPAR-γ-CKO and WT mice (24-28 g) were used in these studies. The animals were provided with ample food and water and maintained on a 12 hr-12 hr light-dark cycle. UUO was performed using an established protocol as previously described [14]. Briefly, all mice received preoperative analgesia (subcutaneous injection of 50 mg/kg buprenorphine), and the right ureter was subsequently ligated with 4.0 surgical silk sutures through a small abdominal incision under 2.0% isoflurane-induced anesthesia. The abdomen was closed in two layers, and the mice were allowed to recover from surgery for 12 hours in a 28°C room. To evaluate the effect of PPAR-γ on renal fibrosis, the mice were randomized into the following four groups (*n* = 6 in each group): (1) sham operated mice of WT mice, (2) UUO model of WT mice, (3) sham operated PT-PPAR-γ-CKO mice, and (4) UUO model of PT-PPAR-γ-CKO mice. All of the mice were euthanized after 14 days of UUO.

### Histological analysis

Two-micrometer sections of paraffin-embedded kidney tissue were subjected to Masson's trichrome staining using commercial kits (Sigma-Aldrich, St. Louis, MO) according to the manufacturer's protocol.

### Western blotting

Cells were grown and stimulated with TGF-β1. After treatment, the cells were washed with PBS and lysed in lysis buffer (20 mM Tris, pH 7.5, 150 mM NaCl, 1% Triton X-100, 1 mM EDTA, 10 μg/ml aprotinin, 10 μg/ml leupeptin, 1 mM phenylmethylsulfonyl fluoride). SDS-PAGE gel was used to separate the proteins. The gel was transferred to a PVDF membrane, which was blocked with 5% non-fat milk in TBST. The blots were probed with a primary antibody followed by a HRP-conjugated secondary antibody. The signal was detected using a Chemiluminescent ECL reagent kit (Millipore).

### Real-time quantitative reverse transcription (qRT-PCR)

The total RNA was isolated from HK-2 cells and the kidney cortex using a Trizol Total RNA Isolation kit (Invitrogen) according to the manufacturer's protocol. The RNA was eluted with RNase-free water. Reverse transcription was performed using the Superscript III RT kit (Invitrogen) according to the manufacturer's protocols. Briefly, the reactions were incubated at 65°C for 5 min and then at 50°C for 60 min. Oligo nucleotides were designed by Primer3 software (available at http://frodo.wi.mit.edu/cgi-bin/primer3/primer3_www.cgi) and synthesized by Invitrogen. Real-time PCR amplification was performed using the SYBR Green master mix (Roche company) and the Prism 7500 Real-time PCR Detection System (Applied Biosystems). The cycling conditions were 95°C for 10 min followed by 40 repeats of 95°C for 15 s and 60°C for 1 min. The relative amounts of mRNA were normalized by GAPDH and calculated using the delta-delta method from threshold cycle numbers.

### DCFDA fluorescence measurement of ROS

The fluorogenic substrate 2′,7′-dichlorofluorescein diacetate (DCFDA) is one of the most widely used techniques for directly measuring the redox state of a cell and could be used to evaluate the intracellular generation of ROS. For measurement of ROS, cells were grown onto glass cover slides. Then, the cells were washed twice with PBS and incubated for 30 min with DCFDA 50 μM when they reached 80% confluence. The cells were then treated with TGF-β1 for 30 min. At the end of the incubation period, the cells were again washed twice with PBS and imaged by confocal laser microscopy. To monitor the ROS levels, cells were seeded into 96-well plates and treated as mentioned above. The relative fluorescence was measured by a fluorescence plate reader (FLUO star OPTIMA) at excitation and emission wavelengths of 485 and 528 nm, respectively, three times at 90-s intervals. The relative fluorescence unit (RFU) was expressed as the fold increase over untreated cells.

### Statistical analysis

All data are shown as the means ±SE. Analysis of variance (ANOVA) was followed by a Bonferroni post-test. A *P* value < 0.05 was considered significant.
